# Zeta potential in oil-water-carbonate systems and its impact on oil recovery during controlled salinity water-flooding

**DOI:** 10.1038/srep37363

**Published:** 2016-11-23

**Authors:** Matthew D. Jackson, Dawoud Al-Mahrouqi, Jan Vinogradov

**Affiliations:** 1 Department of Earth Science and Engineering, Imperial College London, UK; 2 Petroleum Development Oman, Muscat, Oman; 3 Now at the School of Engineering, University of Aberdeen, Aberdeen, UK

## Abstract

Laboratory experiments and field trials have shown that oil recovery from carbonate reservoirs can be increased by modifying the brine composition injected during recovery in a process termed controlled salinity water-flooding (CSW). However, CSW remains poorly understood and there is no method to predict the optimum CSW composition. This work demonstrates for the first time that improved oil recovery (IOR) during CSW is strongly correlated to changes in zeta potential at both the mineral-water and oil-water interfaces. We report experiments in which IOR during CSW occurs only when the change in brine composition induces a repulsive electrostatic force between the oil-brine and mineral-brine interfaces. The polarity of the zeta potential at both interfaces must be determined when designing the optimum CSW composition. A new experimental method is presented that allows this. Results also show for the first time that the zeta potential at the oil-water interface may be positive at conditions relevant to carbonate reservoirs. A key challenge for any model of CSW is to explain why IOR is not always observed. Here we suggest that failures using the conventional (dilution) approach to CSW may have been caused by a positively charged oil-water interface that had not been identified.

Carbonates are the group of rocks composed primarily of the minerals calcite (CaCO_3_) and dolomite (MgCO_3_) along with impurities such as quartz, anhydrite, clay minerals, organic matter and apatite^1^. Carbonate rocks host most of the world’s oil reserves (>60%)^2^. However, the proportion of oil recovered from carbonate reservoirs is low (typically <40%)^3^. Methods for improved oil recovery (IOR) from carbonate reservoirs are therefore of broad scientific interest.

The first (primary) recovery from an oil reservoir results from pressure depletion and is usually modest (5–10%)^4^. In many reservoirs, water is injected to increase (secondary) recovery by maintaining the reservoir pressure and displacing oil towards the production wells. This process is termed water-flooding^5^. The water injected may be formation brine produced from the oil reservoir or an underlying aquifer, seawater, or water from some other convenient source. Formation brine (FMB) is typically highly saline (total ionic strength >2 mol·dm^−3^) and rich in monovalent and divalent ions such as Na^+^, Cl^−^, Ca^2+^ and Mg^2+^ 6,7 (Table 1). Seawater (SW) is less saline than typical FMB (total ionic strength 0.55–0.69 mol·dm^−3^) but is still rich in a range of monovalent and divalent ions^8^,^9^,^10^,^11^ (Table 1).

Laboratory experiments and field trials have shown that oil recovery from carbonate reservoirs can be increased by modifying the composition of the brine injected during water-flooding in a process termed ‘controlled salinity water-flooding’ (CSW)^8^,^9^,^11^,^12^,^13^,^14^,^15^,^16^. In laboratory core-flooding experiments, oil is displaced from cylindrical rock samples (‘cores’) by injecting water of the desired composition into one end face of the core and producing oil (and water) from the opposing face in order to model the reservoir water-flooding process (e.g., Fig. 1a,b,d). In spontaneous imbibition (SI) experiments, core samples are immersed in water and the volume of oil that is spontaneously displaced by water is measured (e.g., Fig. 1c). These laboratory experiments are slow and expensive to undertake, as are field trials^17^. A reliable method for identifying the optimum composition for CSW is therefore essential.

The typical CSW approach in carbonates is to switch from formation brine to seawater^15^,^18^,^19^ (e.g., Fig. 1a) and/or to dilute formation brine or seawater^10^,^20^ (e.g., Fig. 1b). Other studies have adjusted the concentration of one or more of the divalent ions Ca^2+^, Mg^2+^ or SO_4_^2−^ 8,9,13,21,22 (e.g., Fig. 1c). Increases in recovery of up to 20% over standard water-flooding have been observed, comparable with other modern IOR methods^16^,^23^. The problem is that improved recovery is not observed in all cases^16^,^20^ (e.g., Fig. 1d). Despite numerous studies, CSW in carbonates remains poorly understood. Many studies have reported the successful application of CSW, but many others (and more unpublished) observed no benefit, and the available data are often inconsistent and contradictory. More fundamentally, the pore to mineral-surface-scale mechanisms responsible for improved oil recovery during CSW are poorly understood. Consequently, there is no method to predict the optimum CSW composition to maximize oil recovery for a given crude oil/brine/rock (COBR) system.

Improved recovery during CSW in carbonates appears to be observed when the initial wetting state is mixed- or oil-wet, and the wettability changes to become more water-wet after injecting water with modified composition^8^,^9^,^12^,^15^,^21^,^22^,^24^,^25^,^26^. As the reservoir first fills with oil, polar functional groups on the molecules in the oil may adsorb onto the mineral surfaces, rendering them oil-wet. During CSW, some of these molecules desorb, the surfaces become more water-wet and oil is released. Several adsorption/desorption processes at the calcite surface have been proposed^11^,^12^,^21^,^26^,^27^. Many of these cause changes in the zeta potential of the mineral surface. The zeta potential is a measure of the electrical potential in the diffuse (outer) part of the electrical double layer (EDL)^28^ and modifies the electrostatic forces acting between the surface and the polar functional groups in the oil, consistent with the Derjaguin-Landau-Verwey-Overbeek (DLVO) theory^29^.

The zeta potential of calcite depends on the concentration-dependent adsorption of the lattice ions Ca^2+^, Mg^2+^ and CO_3_^2−^ in the Stern layer (the inner part of the EDL)^30^,^31^,^32^,^33^,^34^,^35^,^36^,^37^,^38^,^39^,^40^,^41^. At the high Ca^2+^ and/or Mg^2+^ concentration typical of formation brines, the zeta potential of the calcite surface is positive. Reduction of Ca^2+^ and/or Mg^2+^ concentration, either selectively or by bulk dilution, can invert the polarity, yielding negative zeta potential^40^,^41^,^42^,^43^,^44^,^45^. Addition of SO_4_^2−^ can also yield more negative zeta potential^8^,^30^,^40^,^46^. Several studies have suggested a link between wettability and zeta potential in carbonates, although none have shown a direct correlation between changes in zeta potential, wettability and improved recovery during CSW^21^,^40^,^43^,^47^.

Previous studies have typically utilised measurements of electrophoretic mobility to determine the zeta potential of artificial and natural calcite and carbonate^30^,^31^,^32^,^33^,^34^,^35^,^42^,^43^,^44^,^45^,^46^. In this approach, the sample is crushed to a fine powder and suspended in a solution of the electrolyte of interest. An electrical potential field is applied across the suspension (the field typically oscillates at a controlled frequency, inducing an alternating current through the suspension) and the resulting movement of the solid particles is measured and used to interpret the zeta potential via a suitable version of the Helmholtz-Smoluchowski equation^28^. Commercially available zetameters are available to obtain such data. However, the measurement conditions are far from those employed in CSW experiments for a number of reasons. First, the sample is powdered rather than intact. Second, the temperature and/or total ionic strength are typically lower than in CSW, because hydrocarbon reservoirs are deeply buried and initially contain high ionic strength natural brines. Third, there is no oil present.

The properties of the oil-water interface remain poorly understood. The available data suggest that the interface is negatively charged at pH > 4.5 but can become positive at lower pH^24^,^43^,^48^,^49^,^50^,^51^. However, the data are limited and were acquired using emulsions of oil in simple NaCl or CaCl_2_ electrolytes at low ionic strength, in the commercial zetameters discussed above. It can be challenging to maintain a stable suspension in such experiments, especially in natural brines with high ionic strength^44^,^50^.

Here we show that the zeta potential at both the mineral-water and oil-water interfaces must be determined when assessing the optimum brine composition for CSW in carbonates. We use an integrated experimental apparatus that allows the zeta potential to be interpreted from streaming potential measurements obtained during CSW core-flooding experiments. Our results demonstrate for the first time that the oil-water interface can be positively charged at the high pH and ionic strength relevant to the formation brines found in many carbonate reservoirs. Improved recovery during CSW in carbonates is observed only if the change in brine composition yields a zeta potential at each interface that has the same polarity, such that a repulsive electrostatic force acts between the interfaces and stabilizes a water film on the mineral surface. A key challenge for any model of CSW in carbonates is to explain those cases where no incremental recovery was observed. Here we suggest that failures using the conventional (dilution) approach to CSW, or some other change in water composition that yields a more negative zeta potential at the mineral surfaces, may have been caused by a positive zeta potential at the oil-water interface that had not been diagnosed.

We provide a new method to allow the optimum brine composition for CSW in carbonates to be determined based on measurements of the zeta potential at both the oil-brine and mineral-brine interfaces using an integrated experimental apparatus, along with new insight into the mineral-surface-scale mechanisms underpinning CSW. The results have broad application and significance in allowing oil companies to design water injection strategies that yield improved oil recovery from carbonate reservoirs.

## Methods

Core samples of one limestone, three different brine compositions and four different types of crude oil were used in the experiments reported here (Table 1). The core samples were from the Upper Cretaceous Estaillades limestone, quarried in France (Table 1). The brines were synthetic solutions of reagent-grade NaCl, CaCl_2_, Na_2_SO_4_ and MgCl_2_ salts in deionized water. Three of the brine compositions tested were designed to represent the natural formation brine typical of hydrocarbon reservoirs^6^ (denoted FMB), natural seawater (denoted SW) and seawater diluted 20 times (denoted 20dSW) (Table 1). Similar brines have been used previously in studies of CSW in carbonates^40^,^43^,^45^. The other tested brines comprised two sets each containing three synthetic brines; each set had the same ionic strength but varying Ca^2+^ concentration (Table 1).

Samples saturated with the various brines of interest were prepared using the approach of Alroudhan *et al*.^40^. This approach is designed to ensure equilibrium between the brine and sample at conditions relevant to natural systems, and the enhanced cleaning protocol ensures that divalent ions have been flushed from the mineral surfaces (see the Methods section in the Supplementary Information for additional details). Brine-saturated samples were strongly water-wet.

Samples saturated with brine and crude oil were prepared using the approach of Jadhunandan and Morrow^52^. This approach is designed to replicate the wettability altering reactions that occur in subsurface reservoirs by equilibrating (‘aging’) samples at elevated temperature. The water-saturated samples described above were drained to various values of initial water saturation (*S*_*wi*_) using the crude oil of interest before being aged at 80 °C for four weeks. The wettability obtained after aging depends on *S*_*wi*_ because only those pores occupied by crude oil can have their wettability changed^53^. In some cases, samples were prepared with no water present (*S*_*wi*_ = 0) by saturating clean and dry samples with the crude oil.

The wettability of the samples was determined by measuring the Amott wettability index of water^54^


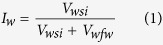


where *V*_*wsi*_ is the volume of water spontaneously imbibed (SI) by the sample and *V*_*wfw*_ is the additional volume of water that can be forced into the sample during water-flooding. The value of *V*_*wsi*_ was measured by placing the sample in a SI cell surrounded by the brine of interest for four weeks. The value of *V*_*wfw*_ was measured using the brine of interest and the core-flooding apparatus shown in Figure S1 of the Supplementary Information. All SI and coreflood tests were conducted at room temperature.

We used the streaming potential method and the apparatus described by Jackson and co-workers^40^,^47^,^55^,^56^; see the Methods section and Figure S1 in the Supplementary Information) to measure the zeta potential of the samples saturated with the various brines and crude oils of interest. The method is applicable to intact natural rock samples, can be used to measure zeta potential at high ionic strength and can also be used for samples saturated with more than one fluid phase. Moreover, the apparatus can be used for core-flooding, so experiments to measure zeta potential and oil recovery were combined here to ensure consistent samples and experimental conditions. The zeta potential was measured on intact samples saturated with brine (*S*_*w*_ = 1), and samples saturated with brine and crude oil at the residual oil saturation (*S*_*w*_ = 1 − *S*_*or*_) after CSW with the brine of interest.

A tertiary CSW approach was adopted in which the core sample was first flooded with the same brine used during aging until no more oil was produced (i.e., the residual oil saturation *S*_*or*_ had been reached, usually after >10 pore-volumes-injected (PVI) of the brine of interest). Thereafter, the injected brine was changed to one with modified composition for a minimum of 10 PVI or until no further oil was produced. Four different water-flooding sequences were used. The first followed a typical low salinity waterflood (LSW): the sample was first aged and then flooded with the formation brine, before switching to seawater and then dilute seawater (FMB → SW → 20dSW). The second inverted the typical LSW: the sample was aged and then flooded with dilute seawater, before switching to seawater and then formation brine (20dSW → SW → FMB).

The third and fourth flooding sequences were designed to test the relative importance of changing the total ionic strength of the injected brine versus changing the concentration of Ca^2+^ which is known to play a key role in controlling the zeta potential of calcite. In the third flooding sequence, the sample was aged and then flooded with the formation brine. Then Ca^2+^ was progressively stripped out of subsequent water-flooding experiments (see compositions FdCa1, FdCa2 and FdCa3 in Table 1) but the ionic strength was kept constant by adjusting the concentration of NaCl. In the fourth flooding sequence, the sample was aged and then flooded with pre-equilibrated NaCl (NaCa0). Then Ca^2+^ was progressively added to subsequent waterfloods (see compositions NaCa1, NaCa2 and NaCa3 in Table 1) whilst keeping the ionic strength constant by adjusting the concentration of NaCl.

## Results

### Brine composition and the zeta potential of water-wet carbonate samples

**Figure 2:**
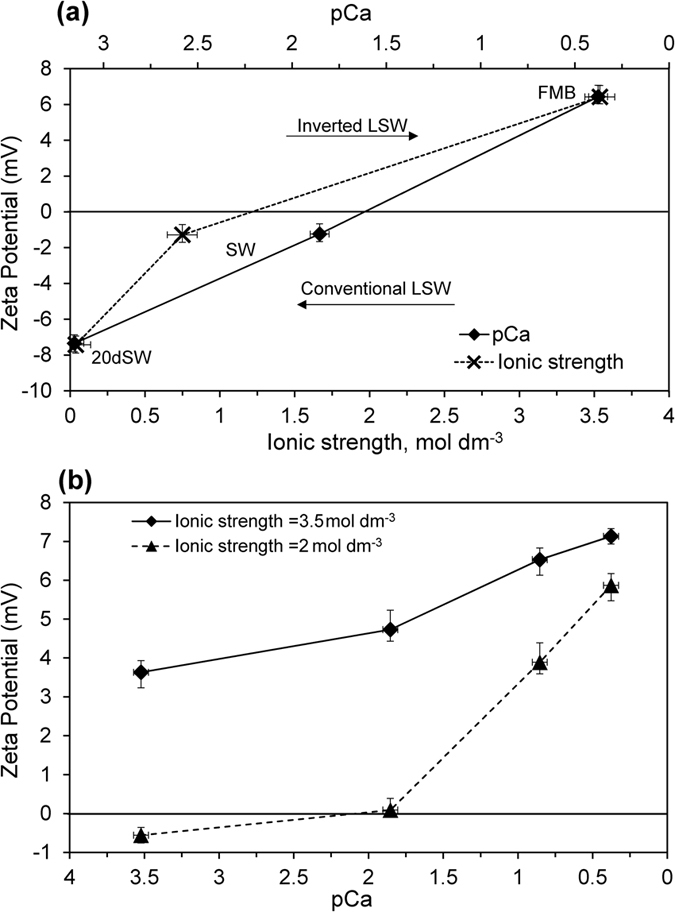
Impact of brine composition on the zeta potential of water-wet carbonate samples. (**a**) Effect of varying composition from formation brine (FMB) to seawater (SW) to dilute seawater (dSW). Data are plotted against ionic strength on the lower horizontal axis and pCa on the upper horizontal axis, where p represents the negative logarithm. (**b**) Effect of varying calcium concentration at two different values of (constant) total ionic strength (2 mol·dm^−3^ and 3.5 mol·dm^−3^ comparable to FMB). Data are plotted against pCa.

We begin by reporting the effect of the tested brine compositions on the zeta potential of strongly water-wet samples saturated only with the brine of interest (Fig. 2). The raw experimental data used to obtain these results are reported in the Results section of the Supplementary Information. The zeta potential of the sample in formation brine was positive, but became negative in contact with seawater, and increasingly negative as the seawater was diluted (Fig. 2a). These results are consistent with previous studies of natural carbonates^40^,^43^,^45^,^47^. Thus a typical low salinity waterflood yields increasingly negative zeta potential at the mineral-brine interfaces. Removing the calcium ions from formation brine also yielded increasingly negative zeta potential, while adding Ca^2+^ to NaCl brine yielded increasingly positive zeta potential (Fig. 2b). However, the change was smaller in magnitude than that observed when switching from formation brine to seawater and then dilute seawater. Moreover, removing Ca^2+^ from the formation brine failed to invert the polarity of the zeta potential.

### Correlation between zeta potential and wettability

**Figure 3:**
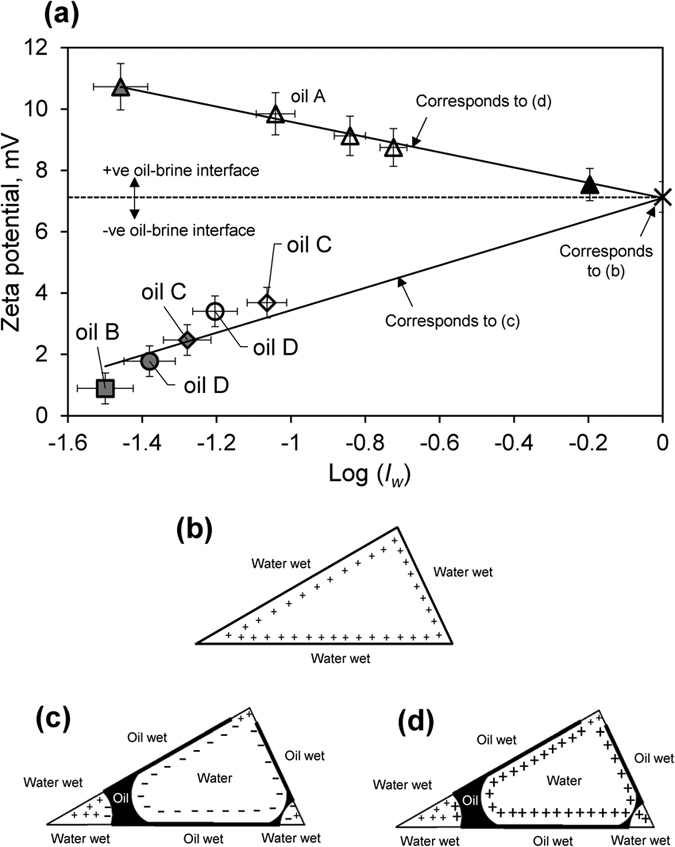
Effect of wettability on zeta potential. (**a**) Zeta potential as a function of water wetting index for each of the four crude oils tested (A–D) aged with FMB. Grey symbols represent aging in the absence of water, open symbols represent aging with water and the filled triangle represents the non-aged sample. (**b,c,d**) Schematics showing a hypothetical triangular pore occupied by (**b**) water and (**c,d**) oil and water. Signs denote the polarity of the zeta potential at water-wet or oil-wet mineral surfaces in, (**b**) oil-wet samples with a positive zeta potential at the mineral-brine and oil-brine interfaces, (**c**) water-wet samples with a positive zeta potential at the mineral-brine interface, and (**d**) oil-wet samples with a negative zeta potential at the oil-brine interface and a positive zeta potential at the mineral-brine interface. Note that the configuration of electrical charge in (**d**) will be stable except for a few nm (approximately one Debye length, corresponding to the local thickness of the diffuse layer) either side of the three-phase (oil-water-rock) contact points where the polarity of the zeta potential changes. The Debye length is only a few nm or tens of nm depending upon the total ionic strength, and the local distribution of electrical charge within the diffuse part of the EDL is shielded from, and therefore not affected by, nearby electrical charge (such as that at a mineral surface or adjacent EDL) over distances greater than one Debye length^28^.

We now investigate the relationship between the zeta potential and wettability of the aged samples, plotting the zeta potential measured after SI and water-flooding of the samples with formation brine, against log(*I*_*w*_) (Fig. 3a). A selection of the raw experimental data used to obtain the zeta potential values shown in Fig. 3a are reported in the Results section of the Supplementary Information. The zeta potential value at *I*_*w*_ = 1 (log *I*_*w*_ = 0) corresponds to that reported in Fig. 2 for the sample saturated only with formation brine with no crude oil present. The zeta potential is positive for this strongly water-wet sample as described above. Two clear and distinct trends in zeta potential with wettability are obtained. In one, observed for Oils B, C and D, the zeta potential becomes increasingly negative with decreasing *I*_*w*_ (i.e., as the samples become less water-wet). In the other, observed for Oil A, the zeta potential becomes increasingly positive with decreasing *I*_*w*_.

Jackson and Vinogradov^47^ observed a positive zeta potential for strongly water-wet carbonate samples that became more negative (less positive) after aging. They argued that a more negative zeta potential in oil-wet conditions reflects a negative zeta potential at the oil-brine interface. Water-wet mineral surfaces return the zeta potential of the mineral-brine interface, which here we found is positively charged in formation brine (Fig. 3b). However, oil-wet mineral surfaces return the zeta potential of the oil-brine interface, rather than the mineral-brine interface. As the sample becomes increasingly oil-wet, the zeta potential is increasingly controlled by the oil-brine interface developed on oil-wet mineral surfaces. When this is negative, the zeta potential of the sample becomes more negative (Fig. 3c). The results obtained here for Oils B, C, and D are therefore consistent with a negative zeta potential at the oil-brine interface. Moreover, the similar trend in zeta potential with wettability observed here suggests that the zeta potential at the oil-brine interface is similar irrespective of the oil composition for these three particular crude oil compositions.

However, Oil A followed a different trend, with the zeta potential becoming increasingly positive as the sample became more oil-wet. We conducted additional experiments to test this trend and found a linear regression provides an excellent fit to the data. We conclude that Oil A must have a positive zeta potential at the oil-brine interface, which is larger in magnitude than the positive zeta potential at the mineral-brine interface (Fig. 3d). Thus the zeta potential of the sample becomes more positive as the proportion of oil-wet mineral surfaces increases. Note that the pH remained above 6 in all experiments (Table 1; Table S1 in the Supplementary Information tabulates all of the data reported).

The next section shows that the polarity of the zeta potential at the oil-brine interface plays a key role in controlling whether improved recovery is observed during CSW. The experiments test the hypothesis that IOR during CSW occurs only when the electrostatic repulsion between the oil-brine and mineral-brine interfaces increases in response to the change in brine composition during water-flooding.

### Controlled salinity water-flooding with varying ionic strength

**Figure 4:**
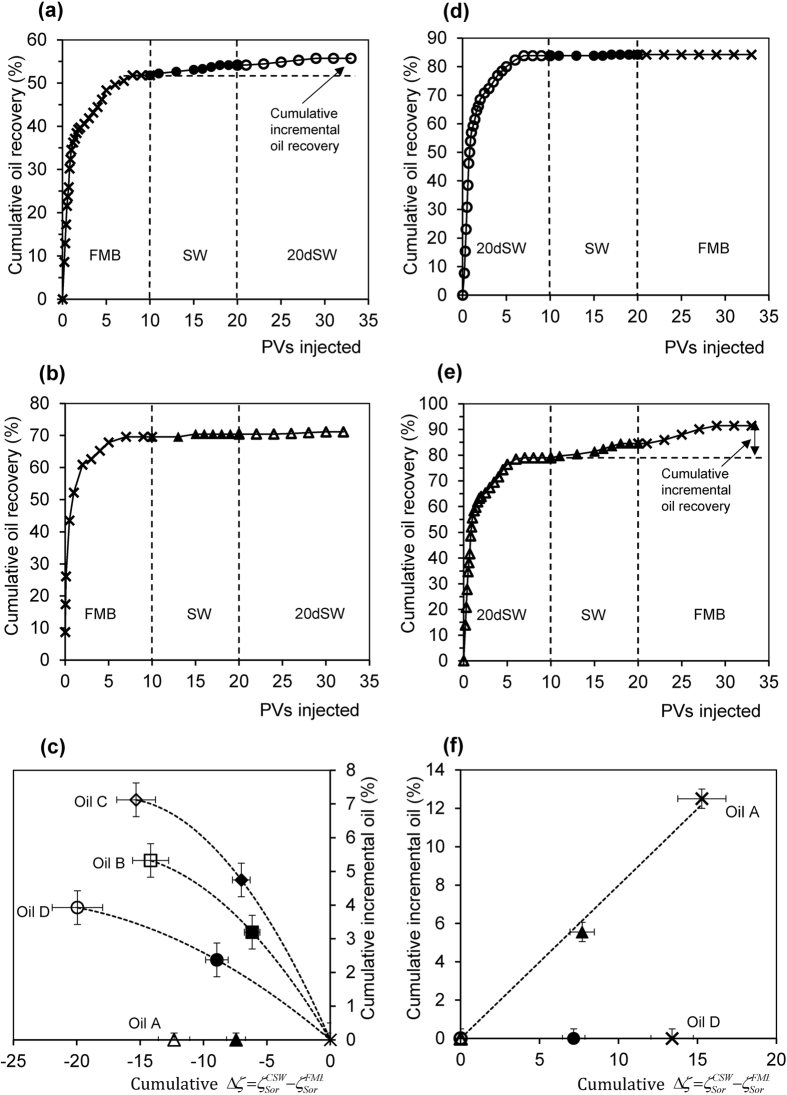
Effect of conventional LSW (FMB → SW → 20dSW) and inverted LSW (20dSW → SW → FMB) on oil recovery and zeta potential change. (**a,b,c**) show conventional LSW results and (**d,e,f**) show inverted LSW results. Examples of (**a**) successful LSW using Crude Oil D and (**b**) unsuccessful LSW using Crude Oil A. Examples of (**d**) unsuccessful inverted LSW using Crude Oil D and (**e**) successful inverted LSW using Crude Oil A. Vertical dashed lines in (**a,b,d,e**) indicate when streaming potential measurements are conducted. (**c,f**) Relationship between incremental oil recovered and the incremental change in the zeta potential of the sample as the injected brine composition was changed. FMB denotes formation brine, SW denotes seawater and 20dSW denotes 20 times diluted seawater.

We now report core-flooding experiments and zeta potential measurements during conventional tertiary LSW (FMB → SW → 20dSW; Fig. 4a,b) and inverted LSW (20dSW → SW → FMB; Fig. 4d,e). As discussed previously, conventional LSW yields more negative zeta potential at the mineral-brine interface; inverted LSW yields more positive zeta potential (Fig. 2a).

All three crude oils B, C and D with a negative zeta potential at the oil-brine interface showed incremental recovery during conventional LSW; the results of one example LSW using Oil D are shown in Fig. 4a. Moreover, there is a clear correlation for each sample between the incremental oil recovered and the incremental change in the zeta potential of the sample as the injected brine composition was changed: oil recovery increased as the zeta potential became more negative (Fig. 4c). By contrast, Oil A showed no incremental recovery during conventional LSW (Fig. 4b) and there is therefore no correlation between change in zeta potential and incremental recovery (Fig. 4c).

During inverted LSW, oils B, C and D showed no incremental recovery; the results of one example inverted LSW using crude Oil D are shown in Fig. 4d. However, Oil A showed the largest incremental recovery of all the crude oils tested (Fig. 4e). Moreover, incremental recovery for Oil A increased as the sample zeta potential became more positive (Fig. 4f). We conclude that CSW is successful only if the zeta potential at the mineral-brine interface changes to become more negative when the oil-brine interface has a negative zeta potential in the formation brine, and *vice-versa*.

### Controlled salinity water-flooding with varying Ca^2+^ concentration

In the conventional and inverted LSW experiments described previously, both the total ionic strength, and the Ca^2+^ concentration, changed between the FMB, SW and 20dSW brines used. We finish by testing the effect of eliminating the change in ionic strength, comparing waterflood experiments in which the Ca^2+^ concentration was progressively decreased at constant ionic strength (FdCa1-3 in Table 1), and in which the Ca^2+^ concentration was progressively increased at constant ionic strength (NaCa1-3 in Table 1). As discussed previously, removal of Ca^2+^ yields more negative zeta potential at the mineral-brine interface; addition of Ca^2+^ yields more positive zeta potential (Fig. 2b).

**Figure 5:**
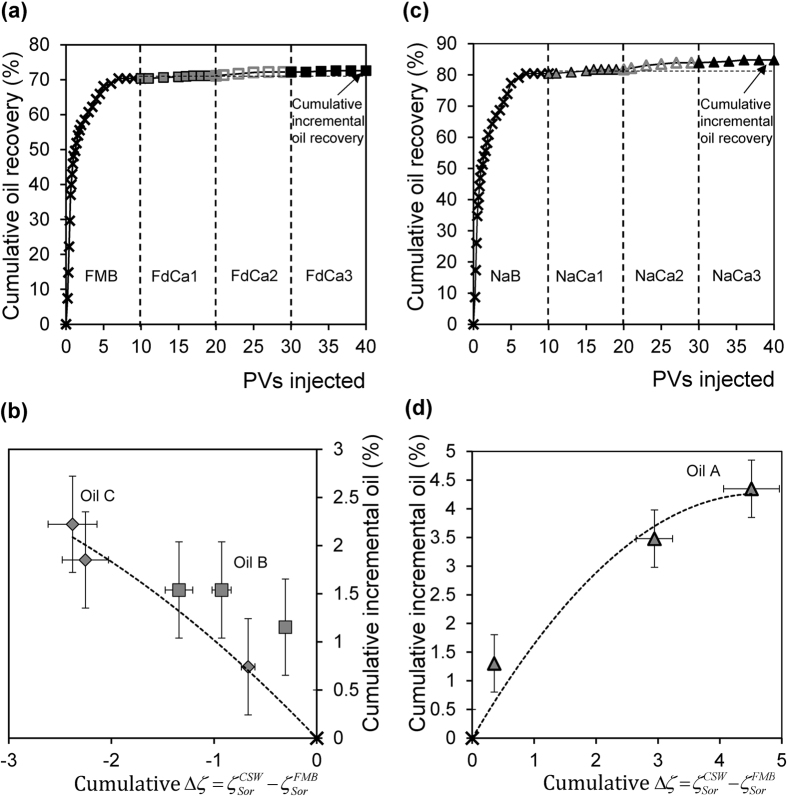
Effect of decreasing/increasing Ca concentration on oil recovery and zeta potential change. (**a**) and (**c**) show examples of incremental recovery when (**a**) Ca is removed during water-flooding of Crude Oil B and (**c**) Ca is added during water-flooding of Crude Oil A. Vertical dashed lines in (**a,c**) indicate when streaming potential measurements are conducted. (**b** and **d**) show the relationship between incremental oil recovered and the incremental change in the zeta potential when (**b**) Ca is removed during water-flooding of Crude Oils B and C, and (**d**) Ca is added during water-flooding of Crude Oil A.

All three crude oils B, C and D with a negative zeta potential at the oil-brine interface showed incremental recovery when Ca^2+^ was removed; the results of one example using Oil C are shown in Fig. 5a. There is again a clear correlation for each sample between the incremental oil recovered and the incremental change in the zeta potential of the sample: oil recovery increased as the zeta potential became more negative (Fig. 5b). By contrast, Oil A showed incremental recovery only when Ca^2+^ was added (Fig. 5c). Incremental recovery for Oil A increased as the sample zeta potential became more positive (Fig. 5d).

We conclude that the composition of the brine is not important so long as CSW causes the appropriate changes in zeta potential at the mineral-brine interface. To observe IOR, the mineral surface must change to have a more negative zeta potential when the oil-brine interface has a negative zeta potential in the formation brine, and *vice-versa*.

### Correlation between zeta potential and improved oil recovery

**Figure 6:**
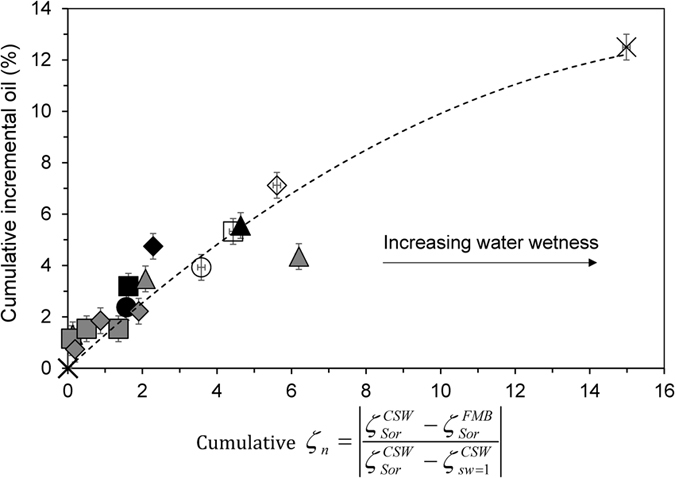
Cumulative incremental oil recovery against cumulative normalised zeta potential for all the CSW experiments showing incremental recovery. Cross shows data in FMB; filled symbols show data in SW and empty symbols show data in 20dSW. Grey symbols show results of decreasing Ca^2+^ (squares for Oil B and diamonds for Oil C) and increasing Ca^2+^ (triangles for Oil A).

Figures 4(c,f) and 5(b,d) show a strong correlation between the measured change in zeta potential of the samples after each change in brine composition and IOR during CSW. However, the trends are sample and oil specific. Here we normalize the change in zeta potential resulting from CSW 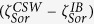 by the change in zeta potential corresponding to wettability alteration 
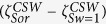
 and plot incremental oil recovery against the incremental normalized change in zeta potential


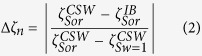


where 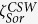 denotes the zeta potential of the aged sample after water-flooding with the chosen brine of controlled composition, 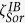 denotes the zeta potential of the aged sample after water-flooding with the initial brine in which the sample was aged, and 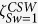 denotes the zeta potential of the sample saturated with the chosen controlled salinity brine at *S*_*w*_ = 1. Plotted in this way, it is clear that incremental recovery correlates with the change in normalized zeta potential induced by CSW irrespective of oil composition or flooding sequence (Fig. 6).

## Discussion

The results presented here demonstrate that the zeta potential at both the mineral-water and oil-water interfaces must be determined when designing the optimum water composition for CSW in carbonates. Improved recovery is observed only if the change in water composition yields a zeta potential at each interface that has the same polarity. If the oil-water interface is negatively charged, modifying the injected water composition to produce a more negative zeta potential on the mineral surfaces yields improved recovery. The conventional approach of diluting formation brine and/or seawater is successful in this case. However, if the oil-water interface is positively charged, the injection water composition must be modified to yield a more positive zeta potential on the mineral surfaces.

**Figure 7:**
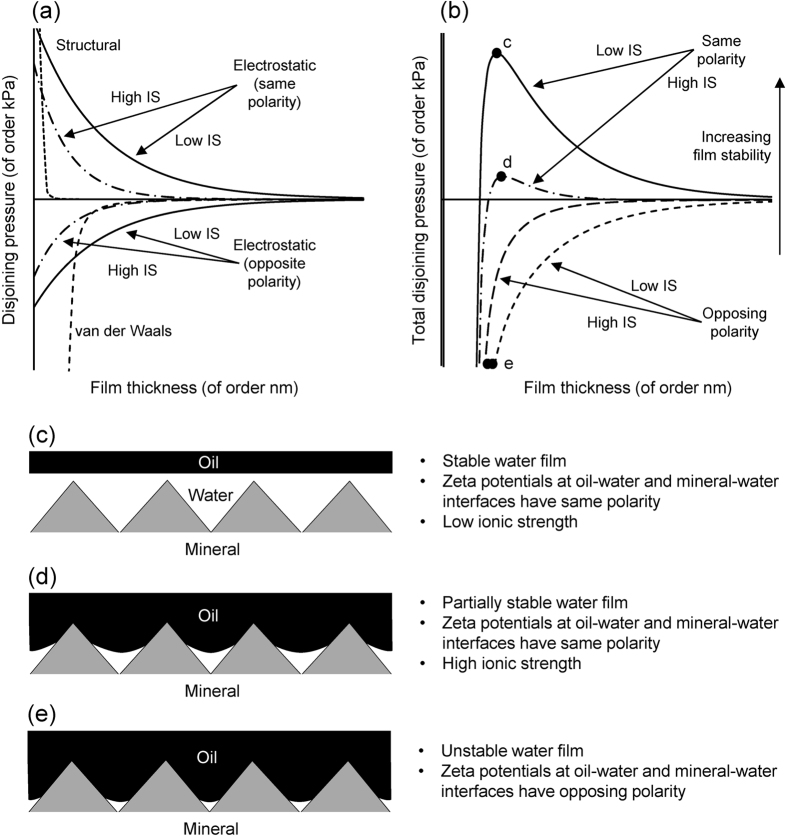
Impact of disjoining pressure on water film thickness and wettability. (**a**) Schematic of the various contributions of van der Waals (dashed line), structural (dotted line) and electrostatic (solid and dot-dash lines) on the disjoining pressure (modified from ref. 57). When the oil-water and mineral-water interfaces have the same polarity, the contribution of electrostatic forces is positive; when they have opposing polarity, the contribution is negative. The contribution of electrostatic forces is larger at low ionic strength (IS) than at high IS. (**b**) Total disjoining pressure corresponding to (**a**). Solid and dot-dash lines in show the disjoining pressure at low and high IS respectively when the interfaces have the same polarity; dot and dash lines show the disjoining pressure at low and high IS when the interfaces have opposing polarity. The labels c-e correspond to figures (**c**–**e**) which show schematically the water film on a rough mineral surface (sketched based on images from ref. 61 and insight from refs 62 and 63). The mineral surfaces are water-wet where the film is stable, but can become oil-wet where the film has collapsed.

The results can be explained at the mineral-surface-scale by considering the stability of a thin water film on the mineral surface that wets and separates the mineral surface from the oil phase^49^,^57^,^58^,^59^,^60^,^61^,^62^,^63^. The stability of a wetting film depends on the disjoining pressure, which is the sum of the contributions from van der Waals forces, structural forces and electrostatic forces^57^ (Fig. 7a). The Van der Waals force is always attractive, which means that its contribution to the disjoining pressure is negative (dashed line in Fig. 7a) and always acts to destabilise the water film. The structural force is always repulsive, so its contribution to the disjoining pressure is positive and always acts to stabilise the water film (dotted line in Fig. 7a).

The contribution of the electrostatic force to the disjoining pressure can be positive or negative depending upon the zeta potential at the mineral-brine and oil-brine interfaces (solid and dot-dash lines in Fig. 7a). If the zeta potentials have the same polarity, then the electrostatic force is repulsive so the contribution of the electrostatic force to the disjoining pressure is positive and *vice-versa* (compare lines above and below the abscissa axis in Fig. 7a). The magnitude of the contribution of the electrostatic force decreases with increasing ionic strength^49^,^59^,^60^ (compare high and low IS lines in Fig. 7a).

The condition for a stable water film is that the disjoining pressure (Π) has a maximum which satisfies^59^


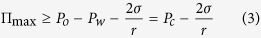


where *P*_*o*_ is the oil pressure (Pa), *P*_*w*_ is the water pressure, *P*_*c*_ is the oil-water capillary pressure, σ is the oil-water interfacial tension (N·m^−1^), and *r* is the local radius of curvature of the mineral surface (m); Fig. 7b). The value of *r* is positive for a concave surface and negative for a convex surface. Thus the maximum disjoining pressure required for water film stability increases with increasing surface roughness and the water film first becomes unstable at an asperity^59^,^61^. When the oil-brine and mineral-brine zeta potentials have the same polarity, the water film can be stable (dark grey lines in Fig. 7b). However, at high ionic strength, only the concave parts of the pore-surfaces host stable water films, whereas at low ionic strength, the water film is stable over a larger proportion of the pore-surfaces. Evidence to support this model comes from nano-scale imaging using cryogenic broad ion-beam polishing in combination with scanning electron microscopy, which has imaged direct contacts between oil and quartz mineral surfaces at asperities in water-wet sandstone samples^61^. When the oil-brine and mineral-brine zeta potentials have opposing polarity, the water film is unstable (pale grey lines in Fig. 7b).

The data obtained here are consistent with the observations that carbonate reservoirs are often mixed-wettability or oil-wet, and that the mineral surfaces in carbonate reservoirs containing typical formation brines rich in Ca^2+^ and Mg^2+^ often have a positive zeta potential. If the oil-brine interface has a negative zeta potential (as in oils B-D investigated here), then the water film is unstable because the zeta potentials at the mineral-brine and oil-brine interfaces have opposing polarity and the electrostatic force is attractive. Consequently, polar components in the oil adsorb onto the mineral surfaces causing the surfaces to become oil-wet (Fig. 7e; location ‘e’ on Fig. 7b). During a conventional LSW, such as that investigated here in which formation brine was replaced by seawater and then dilute seawater, the zeta potential at the mineral-brine interface becomes more negative. Consequently, the electrostatic force becomes repulsive and increases in magnitude, leading to a higher disjoining pressure and increasingly stable water films. Thus the wettability alters towards more water-wet conditions (Fig. 7c; location ‘c’ on Fig. 7b). Oil previously adsorbed on the mineral surfaces is released and incremental oil recovery is observed.

However, if the oil-brine interface has a positive zeta potential (as in Oil A investigated here), the water film is partially stable because the zeta potentials at the mineral-brine and oil-brine interfaces have the same polarity and the electrostatic force is weakly repulsive. Consequently, the polar components in the oil adsorb onto a smaller area of the pore-space (Fig. 7d; location ‘d’ on Fig. 7b). During a conventional LSW, the zeta potential at the mineral brine interface becomes more negative. Consequently, the electrostatic force becomes attractive and increases in magnitude, leading to a lower disjoining pressure and increasingly unstable water films (Fig. 7e; location ‘e’ on Fig. 7b). Thus there is no tendency to re-establish the water wetting layers and no incremental recovery is observed.

In the inverted LSW investigated here, wettability alteration occurred during aging with Crude Oil A and dilute seawater because the attractive electrostatic force between the positively charged oil-brine interface and the negatively charged mineral-brine interface yielded an unstable water film (Fig. 7e; location ‘e’ on Fig. 7b). During CSW, changing the injected brine composition to seawater and then formation brine caused the zeta potential at the mineral-brine interface to become less negative and then positive, leading to increasingly stable water films (Fig. 7d; location ‘d’ on Fig. 7b). Thus the wettability was altered towards more water-wet conditions, oil was released from the mineral surfaces and incremental oil recovery was observed.

Decreasing the Ca^2+^ concentration has a similar effect to simple dilution (and *vice versa*) because varying Ca^2+^ changes the zeta potential of the mineral-brine interface, thus affecting the electrostatic contribution to the disjoining pressure. However, changes in zeta potential in response to changes in Ca^2+^ concentration were less pronounced for the carbonate sample investigated here than the changes in zeta potential observed during conventional and inverted LSW. Thus the IOR was less pronounced.

The experimental method demonstrated here provides a practical approach to determine the optimum brine composition for CSW in carbonates. The first step is to determine the wettability and zeta potential of preserved or aged samples saturated with the crude oil and formation brine of interest, and compare the zeta potential with strongly water-wet samples. Our results, along with previous studies, suggest that IOR during CSW is observed only if the initial wetting state is mixed to oil-wet^18^,^19^,^20^,^26^,^27^.

If the zeta potential of the aged samples is more negative than that of the water-wet samples, then the oil-brine interface is negatively charged and the injection brine should be modified to yield a more negative zeta potential at the mineral-brine interface. Previous studies have shown that this can be achieved by simple dilution or by reducing the concentration of Ca^2+^ and/or Mg^2+^. Addition of SO_4_^2−^ can also yield more negative zeta potential.

If the zeta potential of the aged samples is more positive than that of the water-wet samples, then the oil-brine interface is positively charged and the injection brine should be modified to yield a more positive zeta potential at the mineral-brine interface. Previous studies have shown that this can be achieved by increasing the concentration of Ca^2+^ and/or Mg^2+^.

If the zeta potential of the water-wet and aged samples is the same but wettability alteration has clearly occurred, then the zeta potential of the mineral-brine and oil-brine interfaces is the same within experimental error. If the zeta potential is negative, then the injection brine should be modified to yield a more negative zeta potential at the mineral-brine interface and *vice-versa*.

## Conclusions

The zeta potential at both the mineral-water and oil-water interfaces must be determined when designing the optimum brine composition for CSW in carbonates. The experimental method presented here allows this to be done using intact core samples saturated with the crude oil and brine of interest. Results reported here demonstrate for the first time that the oil-water interface can be positively charged at the high pH and ionic strength relevant to the formation brines found in many carbonate reservoirs. Improved recovery during CSW in carbonates is observed only if the change in brine composition yields a zeta potential at each interface that has the same polarity, such that a repulsive electrostatic force acts between the interfaces and stabilizes a water film on the mineral surface.

If the oil-water interface is negatively charged, modifying the injected water composition to produce a more negative zeta potential on the mineral surfaces yields improved recovery. The conventional approach of diluting formation brine and/or seawater is successful in this case. However, if the oil-water interface is positively charged, the injection water composition must be modified to yield a more positive zeta potential on the mineral surfaces. This can be achieved by increasing the concentration of Ca^2+^ or Mg^2+^ ions. Those studies that have failed to observe improved recovery using the conventional (dilution) approach to CSW, or some other change in water composition designed to yield more negative mineral surfaces, may have been dealing with a positive zeta potential at the oil-water interface that had not been diagnosed.

## Additional Information

**How to cite this article**: Jackson, M. D. *et al*. Zeta potential in oil-water-carbonate systems and its impact on oil recovery during controlled salinity water-flooding. *Sci. Rep.* **6**, 37363; doi: 10.1038/srep37363 (2016).

**Publisher's note:** Springer Nature remains neutral with regard to jurisdictional claims in published maps and institutional affiliations.

## Supplementary Material

Supplementary Information

## Figures and Tables

**Figure 1:**
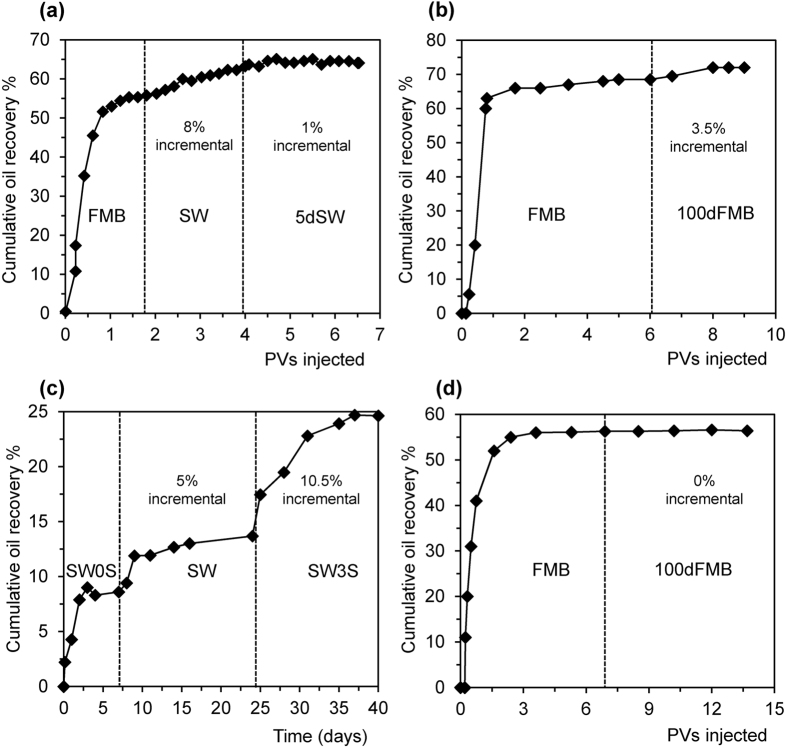
Examples of (**a,b,c**) successful and (**d**) unsuccessful controlled salinity experiments in carbonates. Plots (**a**), (**b**) and (**d**) show core-flooding experiments (data from ref. 15 (**a**) and ref. 20 (**b,d**)). Plot (**c**) shows a spontaneous imbibition (SI) experiment (data from ref. 21). FMB denotes formation brine, SW denotes seawater, 100dFMB denotes formation brine diluted 100 times; 5dSW denotes seawater diluted 5 times; SW0S denotes seawater with SO_4_^2−^ removed; SW3S denotes seawater with SO4^2−^ increased by a factor of 3. Time in plots (**a,b,d**) is denoted in dimensionless pore-volumes (PV) injected.

**Table 1:** Materials used in this study.

Rock samples									
Property/rock					Estaillades				
Description					Upper Cretaceous limestone from France				
Porosity					28% ±0.5				
Permeability					0.13 Darcy ±0.02				
Formation Factor (F)					12.92 ±0.5				
Composition					97% calcite (CaCO_3_), 3% magnesium				
Dimensions					Length (L) = 0.0762 m, Diameter (D) = 0.0381 m				
**Brine samples (molar concentration)**									
	**FMB**	**SW**	**20dSW**	**FdCa1**	**FdCa2**	**FdCa3**	**NaCa1**	**NaCa2**	**NaCa3**
Ions									
Na^+^	2	0.550	0.0275	2.840	3.218	3.259	1.959	1.581	0.741
Cl^−^	3.020	0.620	0.031	3.312	3.428	3.442	1.987	1.861	1.581
Ca^2+^	0.420	0.014	0.0007	0.140	0.014	0.0003	0.014	0.140	0.420
Mg^2+^	0.091	0.045	0.00225	0.091	0.091	0.091	0.00004	0.00004	0.00004
SO_4_^2−^	0.002	0.024	0.0012	0.002	0.002	0.002	0.0002	0.0002	0.0002
Ionic strength	3.537	0.749	0.0375	3.537	3.537	3.537	2.001	2.001	2.001
pH	6.3	7.4	8.2	6.58	6.76	7.24	7.23	6.65	6.29
**Oil samples**									
**Oil Type**	**Acid Number (AN)**			**Base Number (BN)**			**Asphaltene, %**		
Oil A	0.15			0.8			0.05		
Oil B	0.2			1.77			2.9		
Oil C	0.05			0.4			0.1		
Oil D	0.2			1.2			2.3		
