# Supplementary material for: Zeta potential in oil-water-carbonate systems and its impact on oil recovery during controlled salinity water-flooding
Source: Sci Rep. 2016 Nov 23;6:37363. doi: 10.1038/srep37363 (PMC5120358; doi:10.1038/srep37363)
Supplement: Supplementary Information [file srep37363-s1.pdf]

Supplementary Information

Zeta potential in oil-water-carbonate systems and its impact on oil recovery during controlled salinity waterflooding

Matthew D. Jackson[1*], Dawoud Al-Mahrouqi[1, 2] and Jan Vinogradov[1, 3]

[1] Department of Earth Science and Engineering, Imperial College London, UK

[2] Petroleum Development Oman, Oman

[3] Now at the School of Engineering, University of Aberdeen, Aberdeen, UK

Methods

Establishment of initial rock-brine equilibrium

The majority of limestone formations are directly deposited in seawater and the seawater is in equilibrium with both calcite and atmospheric CO_2. After burial, the connate water occupying the pore-space becomes isolated from the atmosphere, and may be replaced by groundwater sourced from elsewhere which equilibrates with the calcite in the absence of atmospheric CO_2. To replicate these natural reservoir conditions, we followed the two stage procedure of Alroudhan et al.[40]. In the first stage of the equilibration procedure, the brine of interest was placed in a beaker with offcuts of the Estaillades limestone, maintaining an air layer in the beaker to provide a source of atmospheric CO_2 but sealing the beaker to prevent evaporation. Monitoring of the pH (using a Five-Go Mettler-Toledo pH meter with their 3-in-1 pH electrode LE438, implementing where necessary the manufacturer's recommended calibration and correction procedures at high ionic strength) and, in selected samples, the Ca^{2+} concentration (using an IC-930 ion chromatographer with appropriate dilution when required) was used to identify when equilibrium had been reached, taken to be when the measured change in both pH and pCa (where p represents the negative logarithm) was zero within experimental error over a timespan of order 100s hours.

The core-flooding apparatus used in the experiments is closed to the atmosphere, and the second stage was to ensure equilibrium between the brine of interest and the rock sample at the closed-system conditions pertaining to a rock-brine system at depth. The rock sample was pre-saturated with the selected brine at open-system conditions and then confined in the core holder at closed-system conditions, and the brine was pumped through the sample from the (closed) inlet reservoir to the (closed) outlet reservoir and back again. The repeated flow of the brine through the sample at closed system conditions mimics migration of the brine into the carbonate rock at depth. At regular intervals, the electrical conductivity and pH of the brine in the reservoirs was measured, and equilibrium was assumed to have been reached when the conductivity and pH of the electrolyte in each reservoir differed by <5%. The electrical conductivity and streaming potential of the brine saturated sample were then measured to determine their values at $S_w = 1$. The sample was then drained with the crude oil of interest to $S_w = S_{wi}$ before being aged at 80ºC for four weeks.

Measurement of streaming potential

The zeta potential was measured using the streaming potential method (SPM) described by Vinogradov et al.[56] and the combined core-flooding and SPM experimental setup shown in Fig. S1. Only a brief summary of the method is provided here. The carbonate core samples were tightly confined within an embedded rubber sleeve in a stainless steel core holder with non-metallic end caps. A syringe pump was used to induce a fluid pressure difference across the sample, causing the fluid of interest (brine during water-flooding and measurements of streaming potential; crude oil during drainage) to flow through the sample from reservoirs connected to each side of the core holder (Figure S1). When injecting brine, synthetic oil was used to translate the induced pressure from the pump to the brine in the inlet reservoir, which maintains closed-system conditions. The

pump maintains constant rate to high accuracy and flow can be directed in either direction through the sample.

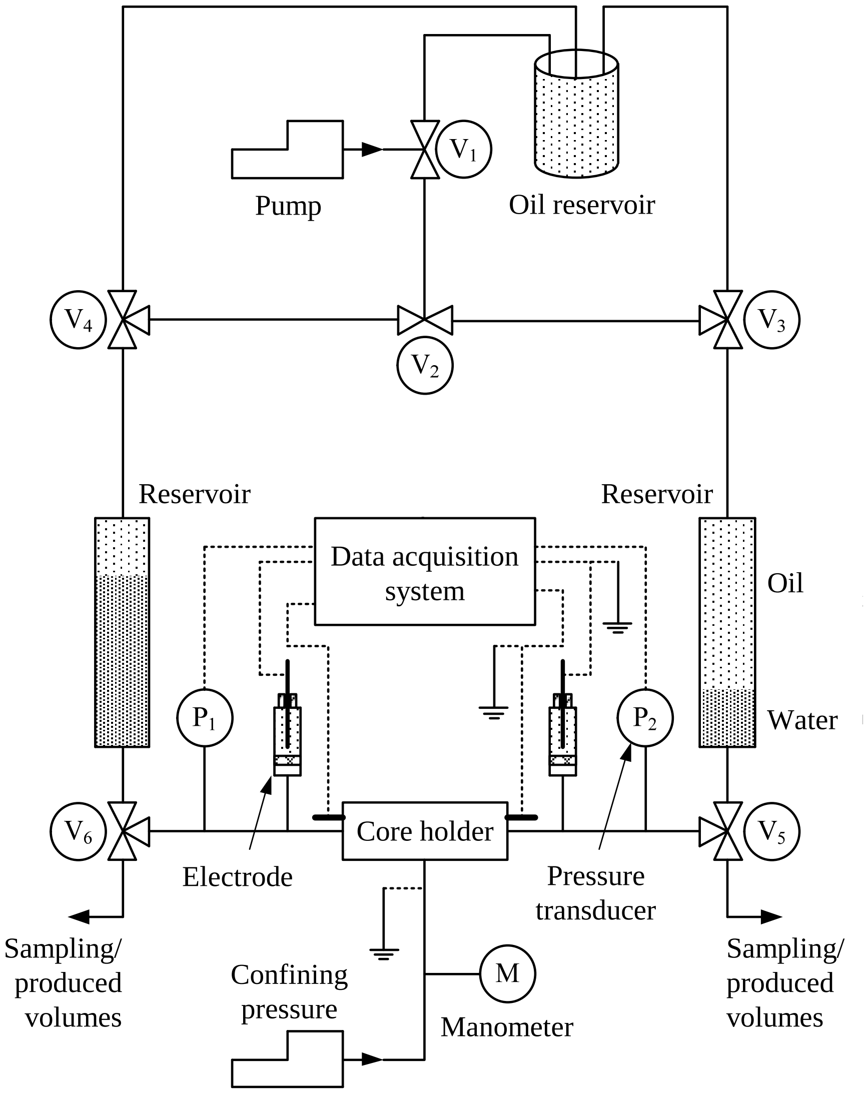

Figure S1. The same experimental setup was used to determine the zeta potential in cylindrical rock cores and to conduct controlled salinity waterflooding experiments. The setup is based on that used by Jackson and co-workers [40,47,55,56]. The setup comprises a pressurized core holder, water/oil reservoirs, pump, flow lines (solid lines) and electrical connections (dashed lines). The flow valves V_1 – V_6 allow the pump to flow water/oil through the core sample in either direction. When injecting water to measure the zeta potential, and in waterflooding experiments, synthetic oil was used to translate the pressure from the pump to the water in the inlet reservoir and maintain closed-system conditions. During drainage, the inlet reservoir was filled with the crude oil of interest. During drainage and waterflooding, produced fluid volumes were measured at the outlet and no attempt was made to correct for dead volumes in the core holder or flow lines. The pressure transducers and electrodes measure the streaming potential ΔV and pressure drop ΔP across the sample.

The pressure difference across the sample was measured using a pair of pressure transducers (calibrated Druck PDCR 810 with accuracy 0.1% of measured value, resolution 70 Pa) and the voltage across the sample was measured using non-polarizing Ag/AgCl electrodes and an NI9219 voltmeter (internal impedance >1GΩ, accuracy 0.18%, resolution 50 nV). The noise level of the measurements is dictated by the stability of the electrodes, rather than the performance of the voltmeter. The electrodes were positioned out of the flow path, in a NaCl electrolyte reservoir of the same ionic strength as the brine used in the experiments.

Note that we did not measure the zeta potential during core-flooding experiments when the oil saturation and, during CSW, the brine composition, were changing within the core sample, because the data are very challenging to obtain and interpret. First, the presence of oil in the inflow (during drainage) or outflow (during water-flooding) lines from the core-holder can isolate the electrodes from the sample, so the electrical potential across the sample cannot be determined (Figure S1). Second, the presence of a compositional gradient in the brine gives rise to an exclusion-diffusion potential across the sample, in addition to the streaming potential signal of interest[SR1]. Removing the contribution of the exclusion-diffusion potential is challenging, as its magnitude and polarity depends upon the pore-scale distribution of the solid-fluid and fluid-fluid interfaces and the charge separation at each interface[SR2]. Instead, the streaming potential data of interest were obtained when only brine was flowing ($S_w = 1$ and $S_w = 1 - S_{or}$).

We used the 'paired-stabilization' or PS method of Vinogradov et al.[56] to measure the streaming potential across the sample, in which flow is induced through the sample at the same rate but in opposing directions. The method eliminates the effect of temporal variations in the static voltage and demonstrates that electrode polarization effects are negligible through confirmation that the change in potential induced by flow in one direction is equal and opposite to the change in potential induced by flow in the opposite direction.

To ensure that exclusion-diffusion potentials were eliminated during measurements of the streaming potential, uniform and constant electrolyte conductivity and pH in each reservoir, and uniform and constant temperature (23°C), were maintained within a 5% tolerance. Redox potentials, which may affect the measured voltage if metals such as steel are in contact with the saline electrolyte, were eliminated by electrically isolating all metallic components from the electrolyte except for the Ag/AgCl electrodes.

Interpretation of the results from the PS experiments followed from the observation that at steady-state, the streaming current induced by the flow is balanced by a conduction current to maintain overall electrical neutrality. At steady-state (when the total current density j=0) the coupling coefficient C is given by [e.g. 56]

$$C = \left.\frac{\Delta V}{\Delta P}\right|_{j=0} \qquad \text{(S1)}$$

The zeta potential was determined using a modified version of the Helmholtz-Smoluchowski equation that accounts for surface electrical conductivity [e.g. 56]

$$\zeta = \frac{C\mu_w \sigma_{rw} F}{\varepsilon_w} \qquad \text{(S2)}$$

where F is the formation factor (the ratio of the saturated rock electrical conductivity to the water electrical conductivity, measured when surface electrical conductivity is negligible; see Table 1), σ_{rw} is the electrical conductivity of the saturated rock ($S \cdot m^{-1}$, and ε_w and μ_w are the permittivity ($F \cdot m^{-1}$) and viscosity (Pa·s) of the water respectively.

The formation factor and electrical conductivity were measured following the methodology of Vinogradov et al.[56] (Table 1). The viscosity and permittivity of the electrolyte as a function of ionic strength were also determined using the approach of Vinogradov et al.[56]. Uncertainty in the reported value of zeta potential reflects the range of possible regressions that can be fitted to the measured streaming potential data within experimental error, as discussed in the following section.

Results

Typical raw results from streaming potential experiments with $S_w = 1$ are shown in Fig. S2. These particular experiments were used to determine the relationship between brine composition and zeta potential in brine-saturated samples (see Fig. 2a in the Main Paper). Each plot in Fig. S2 shows a single PS experiment, with flow induced through the sample in one direction and then the other at a given flow rate. The measured pressure and voltage responses are shown as a function of time elapsed. Flow is induced in a given direction until a stable pressure and voltage are recorded. The stable values for a given flow direction and flow rate are plotted as a single data point on a cross-plot of voltage against pressure difference.

Figure S3 shows the cross-plots corresponding to each value of zeta potential reported in Figure 2a in the Main Paper. The error bars on these cross-plots represent the spread of stable values of pressure and voltage that can be fitted to the raw data. The spread of pressure values is normally within the symbol size. The PS experiments are repeated at a number of different flow rates (typically four) and a linear regression through the plotted values of stable pressure and voltage yields the coupling coefficient (C) from which the zeta potential is interpreted via equation (S2).

Typical raw results from streaming potential experiments with $S_w = 1 - S_{or}$ are shown in Figure S4. These particular experiments were used to determine the relationship between wettability and zeta potential in samples saturated with FMB and Crude Oil A (see Figure 3a in the Main Paper). Figure S5 shows the cross-plots corresponding to four of the values of zeta potential reported for Crude Oil A in Fig. 3a in the Main Paper. Data from the numerous other streaming potential experiments conducted are similar to these examples. The key results are summarized in Table S1.

References specific to the Supplementary Information

SR1 Leinov E. and Jackson M.D., Experimental measurements of the SP response to concentration and temperature gradients in sandstones with application to subsurface geophysical monitoring, *J. Geophys. Res.* **119** doi:10.1002/2014JB011249 (2014).

SR2 Westermann-Clark, G. B. and C. C. Christoforou. The exclusion-diffusion potential in charged porous membranes, *J. Electroanal. Chem.*, **198**, 213–231, doi:10.1016/0022-0728(86)90001-X (1986).

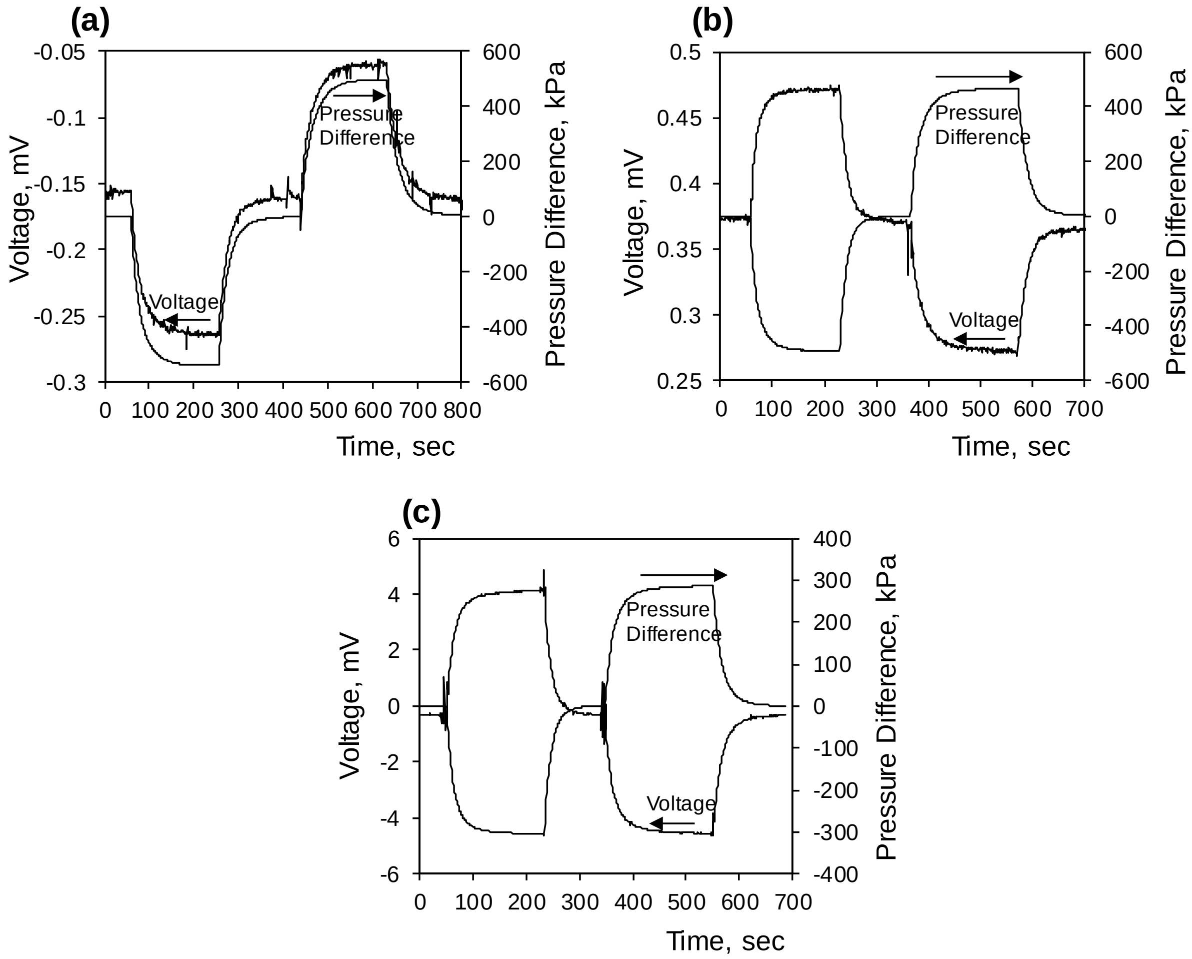

Figure S2. Typical raw results from streaming potential experiments with $S_w = 1$ in (a) FMB, (b) SW, and (c) 20dSW. Each plot shows a single PS experiment, with flow induced through the sample in one direction and then the other. The measured pressure and voltage responses are shown as a function of time. Flow is induced in a given direction until a stable pressure and voltage are recorded. The stable values are plotted as a single data point on a cross-plot of voltage against pressure difference (see Fig. S3).

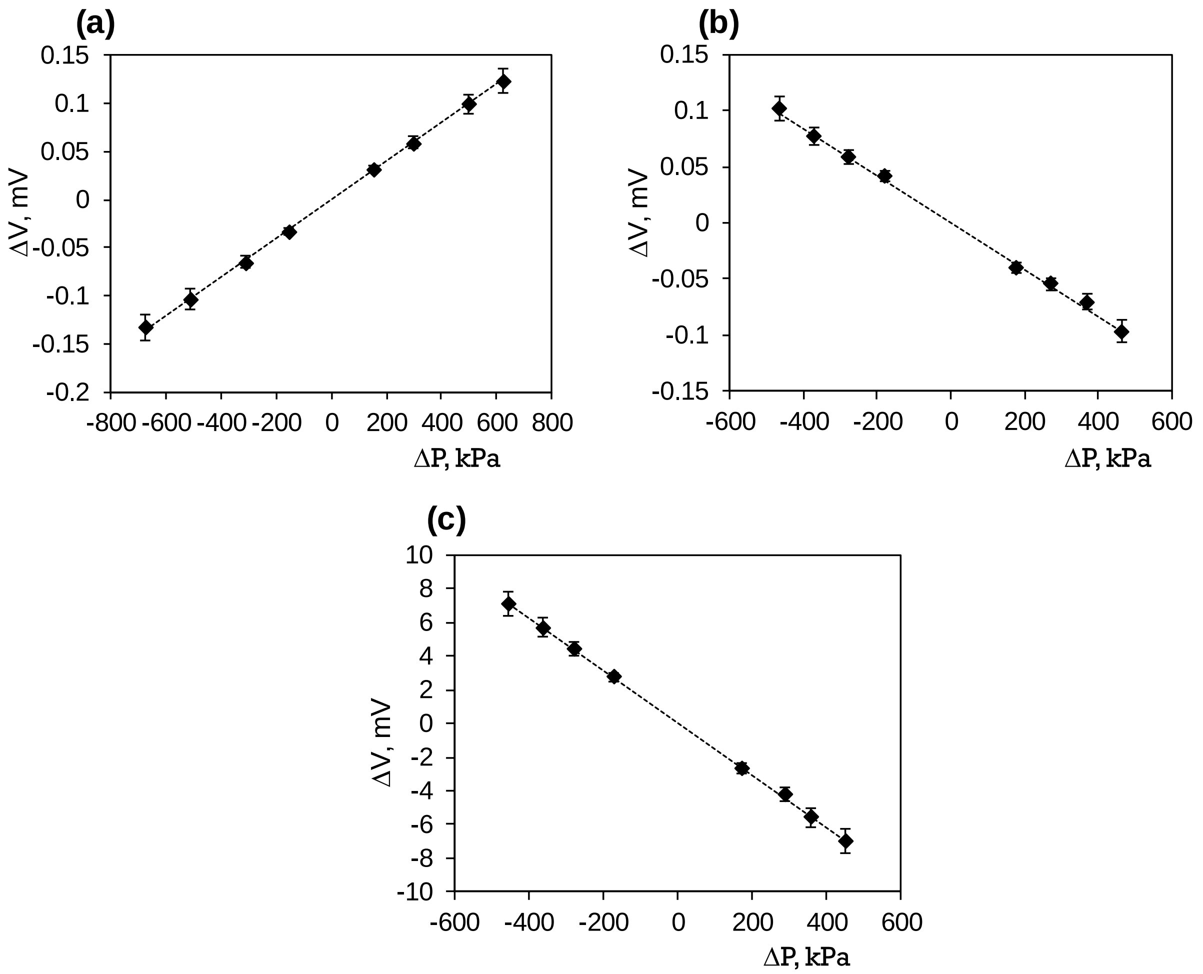

Figure S3. Stabilized voltage vs. stabilised pressure difference from streaming potential experiments with S_w = 1 in (a) FMB, (b) SW and (c) 20dSW corresponding to each value of zeta potential reported in Fig. 2a in the Main Paper.

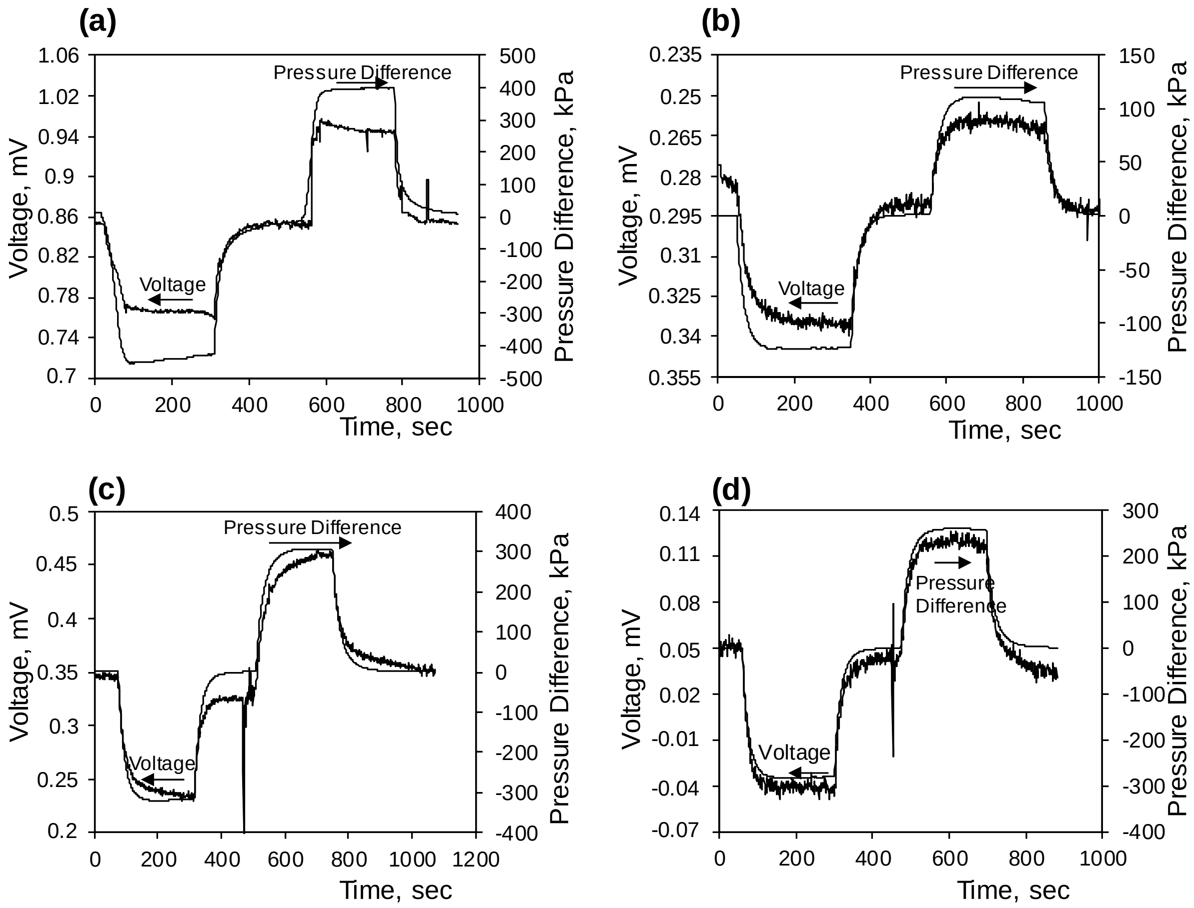

Figure S4. Typical raw results from streaming potential experiments with S_w = 1 - S_{or}. Each plot shows a single PS experiment, with flow induced through the sample in one direction and then the other. The measured pressure and voltage responses are shown as a function of time. Flow is induced in a given direction until a stable pressure and voltage are recorded. The stable values are plotted as a single data point on a cross-plot of voltage against pressure difference (see Fig. S5). These experiments were used to determine the relationship between wettability and zeta potential in samples saturated with FMB and Crude Oil A (see Fig. 3a in the main paper). Data are shown for different values of the Amott water index: (a) I_w = 0.19, (b) I_w = 0.14, (c) I_w = 0.09 and (d) I_w= 0.03.

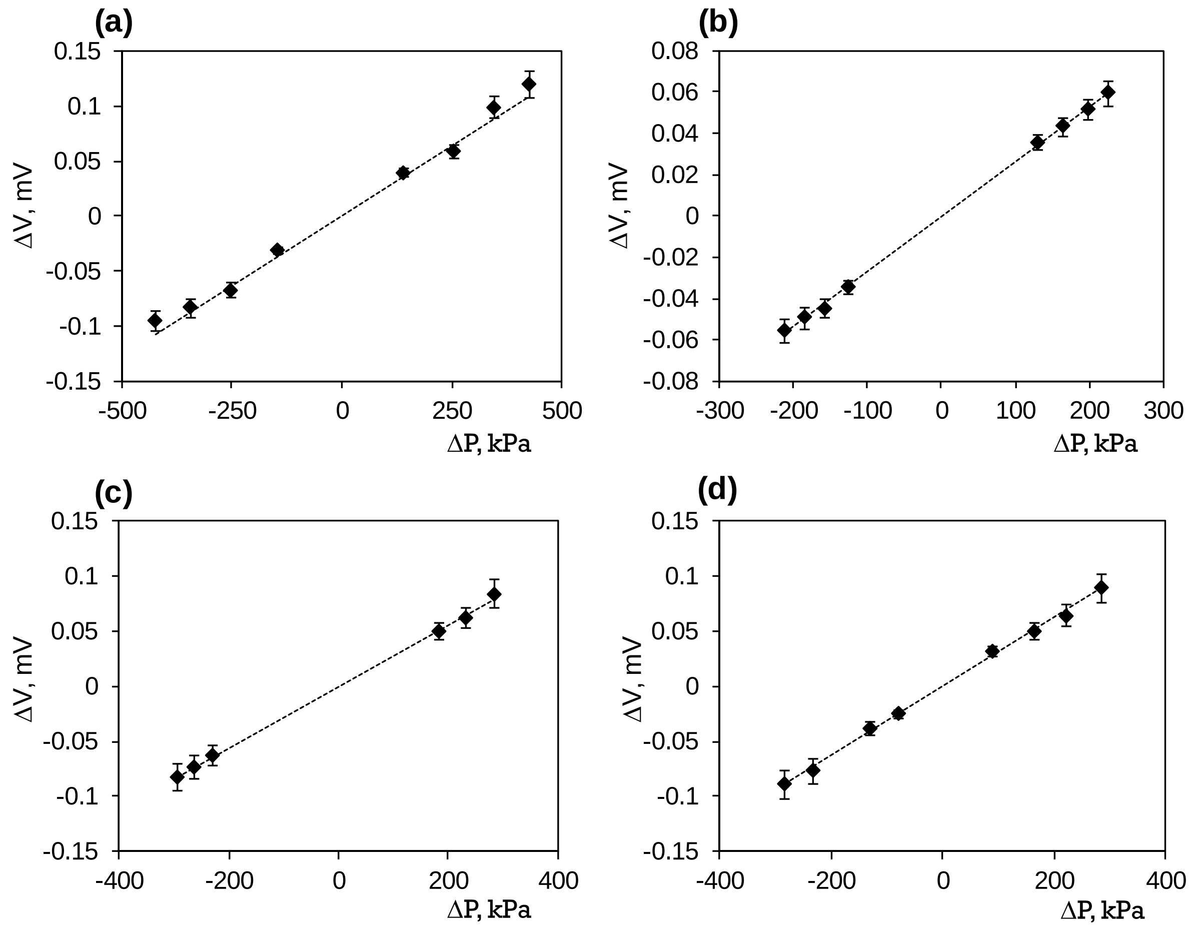

Figure S5. Stabilized voltage vs. stabilised pressure difference from streaming potential experiments with S_w = 1 - S_{or} corresponding to four of the values of zeta potential reported for Crude Oil A in Fig. 3a in the Main Paper. Data are shown for different values of Amott water index: (a) I_w = 0.19, (b) I_w = 0.14, (c) I_w = 0.09 and (d) I_w= 0.03.

Table S1: Compilation of SPM results. Here σ_w denotes the electrical conductivity of the equilibrated brine, and k denotes the permeability of the plug (1D=9.869233×$10^{-13}$$m^2$). The first three rows show experiments at S_w = 1; the remaining rows show experiments at S_w = 1- S_{or}. Brine compositions correspond to those shown in Table 1 and associated text in the Main Paper.

Plug #	Crude Oil	Initial Brine	Flooding Brine	S_{wi}	1-S_{or}	I_w	C (V/Pa)	σ_{rw} (S/m)	σ_w (S/m)	F	ε_w (F/m)	μ_w (Pa·s)	ζ (mV)	k (D)	pH
Ew1	N/R	FMB	FMB	1	1	1	2.001E-10	1.281	14.690	11.471	4.912E-10	1.191E-03	7.131	0.113	6.3
Ew1	N/R	SW	SW	1	1	1	-2.088E-10	0.343	4.515	13.164	6.475E-10	9.957E-04	-1.483	0.123	7.4
Ew1	N/R	20dSW	20dSW	1	1	1	-1.556E-08	0.029	0.379	12.889	7.048E-10	9.483E-04	-8.241	0.121	8.2
Ec1	oil A	FMB	FMB	0.570	0.800	0.640	2.292E-10	0.795	13.900	17.484	4.982E-10	1.179E-03	7.536	0.040	6.74
Ec2	oil A	FMB	FMB	0.670	0.860	0.190	2.550E-10	0.505	14.400	28.540	4.962E-10	1.182E-03	8.748	0.012	6.51
Ec3	oil A	FMB	FMB	0.710	0.920	0.140	2.660E-10	0.490	14.400	29.397	4.962E-10	1.182E-03	9.126	0.010	6.51
Ec4	oil A	FMB	FMB	0.350	0.860	0.090	2.861E-10	0.183	14.440	79.067	4.962E-10	1.182E-03	9.611	0.022	6.51
Ec5	oil A	FMB	FMB	0.000	0.500	0.030	3.128E-10	0.196	14.390	73.322	4.962E-10	1.182E-03	10.726	0.075	6.51
Ec1	oil A	FMB	SW	0.570	0.800	0.640	-2.087E-10	0.146	4.580	31.329	6.522E-10	9.918E-04	-1.454	0.025	7.84
Ec2	oil A	FMB	SW	0.670	0.860	0.190	1.097E-10	0.215	4.437	20.682	6.489E-10	9.945E-04	0.746	0.032	7.40
Ec3	oil A	FMB	SW	0.710	0.920	0.140	1.905E-10	0.097	4.533	46.930	6.472E-10	9.959E-04	1.329	0.038	7.50
Ec4	oil A	FMB	SW	0.350	0.860	0.090	3.495E-10	0.097	4.533	46.930	6.472E-10	9.959E-04	2.438	0.038	7.60
Ec5	oil A	FMB	SW	0.000	0.500	0.030	5.018E-10	0.055	4.636	84.493	6.522E-10	9.918E-04	3.538	0.061	7.84
Ec1	oil A	FMB	20dSW	0.570	0.800	0.640	-1.616E-08	0.013	0.363	28.975	7.042E-10	9.488E-04	-7.910	0.025	7.65
Ec2	oil A	FMB	20dSW	0.670	0.860	0.190	-9.578E-09	0.019	0.407	21.039	7.037E-10	9.492E-04	-5.259	0.032	8.20
Ec3	oil A	FMB	20dSW	0.710	0.920	0.140	-6.700E-09	0.010	0.428	43.034	7.035E-10	9.494E-04	-3.873	0.052	8.31
Ec4	oil A	FMB	20dSW	0.350	0.860	0.090	-4.637E-09	0.010	0.428	43.034	7.035E-10	9.494E-04	-2.680	0.052	8.31
Ec5	oil A	FMB	20dSW	0	0.500	0.030	-2.948E-09	0.006	0.471	76.014	7.037E-10	9.492E-04	-1.871	0.059	8.20
Ec6	oil B	FMB	FMB	0	0.471	0.032	2.565E-11	0.043	14.510	335.480	4.957E-10	1.183E-03	0.888	0.030	6.49
Ec6	oil B	FMB	SW	0	0.503	0.030	-8.064E-10	0.020	4.267	212.382	6.489E-10	9.945E-04	-5.273	0.034	7.41
Ec6	oil B	FMB	20dSW	0	0.524	0.028	-3.013E-08	0.004	0.328	86.326	7.045E-10	9.485E-04	-13.286	0.048	8.31
Ec7	oil C	FMB	FMB	0	0.492	0.053	7.020E-11	0.064	14.590	227.723	4.929E-10	1.188E-03	2.469	0.019	6.51
Ec7	oil C	FMB	SW	0	0.539	0.048	-7.246E-10	0.028	4.222	150.473	6.516E-10	9.923E-04	-4.659	0.019	7.29
Ec7	oil C	FMB	20dSW	0	0.563	0.046	-2.817E-08	0.002	0.339	142.851	7.045E-10	9.485E-04	-12.850	0.017	8.31
Ec8	oil D	FMB	FMB	0	0.518	0.042	5.236E-11	0.072	14.310	199.397	4.977E-10	1.179E-03	1.775	0.049	6.51
Ec8	oil D	FMB	SW	0	0.542	0.040	-1.086E-09	0.035	4.321	123.018	6.502E-10	9.934E-04	-7.171	0.054	7.31
Ec8	oil D	FMB	20dSW	0	0.557	0.039	-4.220E-08	0.003	0.320	118.776	7.046E-10	9.485E-04	-18.170	0.041	8.31
Ec9	oil A	20dSW	20dSW	0.382	0.947	0.023	-2.070E-08	0.002	0.345	193.981	7.037E-10	9.492E-04	-9.624	0.031	8.41
Ec9	oil A	20dSW	SW	0.382	0.904	0.025	-2.814E-10	0.041	4.455	108.977	6.483E-10	9.950E-04	-1.924	0.030	7.30
Ec9	oil A	20dSW	FMB	0.382	0.870	0.026	1.703E-10	0.105	14.150	134.378	5.005E-10	1.175E-03	5.654	0.030	6.51
Ec10	oil D	20dSW	20dSW	0.418	0.906	0.092	-1.168E-08	0.007	0.490	65.677	7.029E-10	9.500E-04	-7.728	0.018	8.41
Ec10	oil D	20dSW	SW	0.418	0.906	0.092	-3.727E-10	0.090	4.479	49.495	6.480E-10	9.953E-04	-2.569	0.019	7.30
Ec10	oil D	20dSW	FMB	0.418	0.906	0.092	1.014E-10	0.283	14.820	52.296	4.890E-10	1.196E-03	3.666	0.016	6.51
Ec11	oil A	NaCa0	NaCa0	0.422	0.887	0.032	9.867E-11	0.232	12.000	51.759	5.374E-10	1.115E-03	2.455	0.043	8.41
Ec11	oil A	NaCa0	NaCa1	0.422	0.894	0.032	1.221E-10	0.228	11.430	50.178	5.470E-10	1.101E-03	2.810	0.041	8.20
Ec11	oil A	NaCa0	NaCa2	0.422	0.907	0.031	2.419E-10	0.175	11.210	63.995	5.507E-10	1.096E-03	5.398	0.045	7.30
Ec11	oil A	NaCa0	NaCa3	0.422	0.912	0.031	3.787E-10	0.178	9.856	55.326	5.725E-10	1.069E-03	6.966	0.043	6.30
Ec12	oil B	FMB	FMB	0.423	0.871	0.059	9.949E-11	0.145	14.310	98.506	4.962E-10	1.182E-03	3.402	0.006	6.51
Ec12	oil B	FMB	FdCa1	0.423	0.880	0.058	7.774E-11	0.249	15.530	62.249	4.769E-10	1.219E-03	3.093	0.008	6.51
Ec12	oil B	FMB	FdCa2	0.423	0.880	0.058	5.875E-11	0.249	15.970	64.012	4.697E-10	1.234E-03	2.471	0.010	6.80
Ec12	oil B	FMB	FdCa3	0.423	0.878	0.059	4.873E-11	0.293	16.010	54.607	4.697E-10	1.234E-03	2.054	0.011	7.30
Ec13	oil C	FMB	FMB	0.459	0.840	0.095	1.069E-10	0.232	14.440	62.243	4.955E-10	1.183E-03	3.687	0.057	6.51
Ec13	oil C	FMB	FdCa1	0.459	0.852	0.092	7.237E-11	0.312	15.920	50.978	4.705E-10	1.233E-03	3.018	0.066	6.51
Ec13	oil C	FMB	FdCa2	0.459	0.850	0.092	3.843E-11	0.337	15.050	44.720	4.853E-10	1.203E-03	1.434	0.073	6.80
Ec13	oil C	FMB	FdCa3	0.459	0.844	0.094	2.944E-11	0.210	16.420	78.077	4.622E-10	1.251E-03	1.340	0.070	7.30
